# ADVANCING PERSON-CENTERED CARE PLANNING FOR PERSONS WITH MULTIPLE CHRONIC CONDITIONS

**DOI:** 10.1093/geroni/igae098.1859

**Published:** 2024-12-31

**Authors:** Ana Quiñones, David Dorr, Arlene Bierman

**Affiliations:** Chair; Co-Chair; Discussant

## Abstract

Providing high quality, comprehensive, longitudinal, person-centered care for persons living with, or at risk for, multiple chronic conditions (MCC) is a critical challenge facing our healthcare system. Persons with MCC often navigate complicated and fragmented healthcare systems that can be inefficient, duplicative, costly, and poorly coordinated which increases risk for avoidable adverse events, and unduly burdens persons with MCC, families, and caregivers with the added responsibility of sharing information and coordinating care across providers. Person-centered care planning (PCCP) is a process of active collaboration and shared decision-making among providers, persons seeking care, families, and caregivers to develop a, person-centered management plan, incorporating patient values, goals, and preferences. This symposium will share preliminary findings of a collaborative, multi-disciplinary AHRQ-funded initiative which aims to examine the current state of the field and convene frontline providers, health care leaders, and policy makers to identify solutions including the necessary resources, conditions, processes, and implementation strategies to address barriers to delivering appropriate, equitable, and high-quality PCCP as a routine component of care delivery. The first presentation will summarize the state of the field regarding PCCP models for persons with MCC identified through environmental scans. The second will discuss barriers, facilitators, and strategies identified during the Partner Roundtable discussions to improve PCCP design and implementation within health care systems. The final presentation will share successes and failures that health professionals and front-line providers have experienced implementing PCCP, as well as best practices and strategies identified during the Learning Collaborative to deliver high-quality PCCP for persons with MCCs.

